# Author Correction: Lineage-specific RUNX2 super-enhancer activates MYC and promotes the development of blastic plasmacytoid dendritic cell neoplasm

**DOI:** 10.1038/s41467-019-11919-x

**Published:** 2019-08-28

**Authors:** Sho Kubota, Kenji Tokunaga, Tomohiro Umezu, Takako Yokomizo-Nakano, Yuqi Sun, Motohiko Oshima, Kar Tong Tan, Henry Yang, Akinori Kanai, Eisaku Iwanaga, Norio Asou, Takahiro Maeda, Naomi Nakagata, Atsushi Iwama, Kazuma Ohyashiki, Motomi Osato, Goro Sashida

**Affiliations:** 1 Laboratory of Transcriptional Regulation in Leukemogenesis, International Research Center for Medical Sciences (IRCMS), Kumamoto University, 2-2-1 Honjo, Chuo Ward, Kumamoto, 860-0811 Japan; 2 Department of Hematology, Kumamoto University, 1-1-1 Honjo, Chuo Ward, Kumamoto, 860-8556 Japan; 3 Department of Hematology, Tokyo Medical University, 6-7-1 Nishi-Shinjuku, Shinjuku, Tokyo, 160-0023 Japan; 4 Department of Cellular and Molecular Medicine, Chiba University, 1-8-1 Inohana, Chuo Ward, Chiba, 260-8670 Japan; 5 Division of Stem Cell and Molecular Medicine, Center for Stem Cell Biology and Regenerative Medicine, The Institute of Medical Science, The University of Tokyo, 4-6-1 Shirokanedai, Minato, Tokyo, 108-8639 Japan; 6 Cancer Science Institute of Singapore, National University of Singapore, Singapore, 119077 Singapore; 7 Department of Molecular Oncology, Research Institute for Radiation Biology and Medicine, Hiroshima University, Hiroshima, 739-0046 Japan; 8 Department of Hematology, International Medical Center, Saitama Medical University, Saitama, 350-1298 Japan; 9 Department of General Medicine, Nagasaki University, Graduate School of Biomedical Science, Nagasaki, 852-8523 Japan; 10 Division of Reproductive Engineering, Center for Animal Resources and Development (CARD), Kumamoto University, 2-2-1 Honjo, Chuo Ward, Kumamoto, 860-0811 Japan; 11 Laboratory of Runx Biology, International Research Center for Medical Sciences (IRCMS), Kumamoto University, 2-2-1 Honjo, Chuo Ward, Kumamoto, 860-0811 Japan; 12 Center for Metabolic Regulation of Healthy Aging (CMHA), Kumamoto University, Chuo Ward, Kumamoto, 860-0811 Japan

**Keywords:** Cancer models, Targeted therapies, Acute myeloid leukaemia, Mechanisms of disease, Cancer epigenetics

Correction to: *Nature Communications* 10.1038/s41467-019-09710-z, published online 10 April 2019.

The original version of this Article contained an error in the spelling of the author Kar Tong Tan, which was incorrectly given as Kar Ton Tan. This has now been corrected in both the PDF and HTML versions of the Article.

