# Supplementary material for: Lineage-specific RUNX2 super-enhancer activates MYC and promotes the development of blastic plasmacytoid dendritic cell neoplasm
Source: Nat Commun. 2019 Apr 10;10:1653. doi: 10.1038/s41467-019-09710-z (PMC6458132; doi:10.1038/s41467-019-09710-z)
Supplement: Supplementary file 1 — Supplementary Information [file 41467_2019_9710_MOESM1_ESM.pdf]

Supplementary Information

Lineage-specific RUNX2 super-enhancer activates MYC and promotes the development of blastic plasmacytoid dendritic cell neoplasm

Kubota et al.

Supplementary Figure 1-16

Supplementary Table 1-2

Supplementary Figure 1

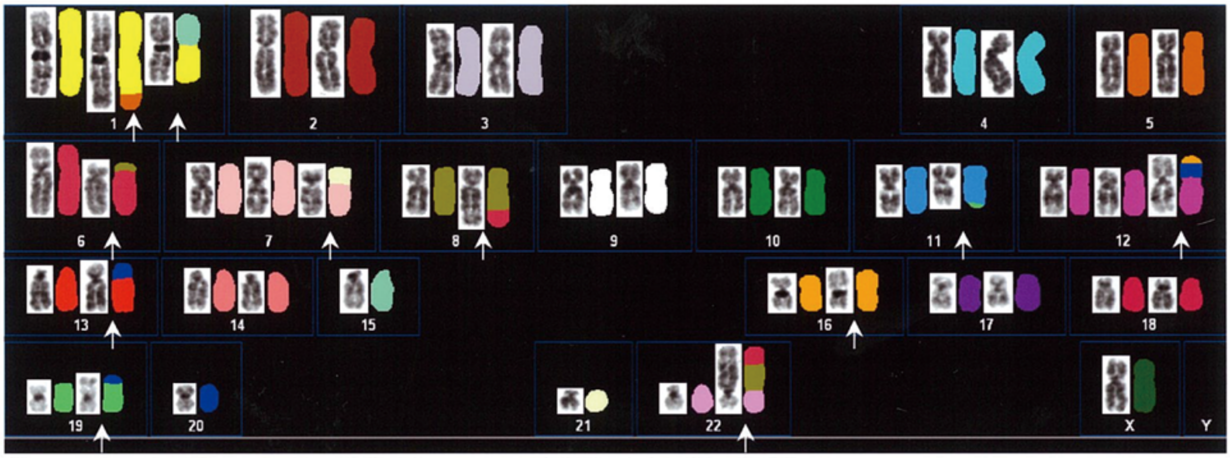

Supplementary Figure 1. Spectral Karyotyping (SKY) analysis of CAL-1 cells. CAL-1 cells harbored chromosomal anomalies of t(6;8) and derivative(22) containing a duplication of the long arm of der(8) identified by SKY.

Supplementary Figure 2

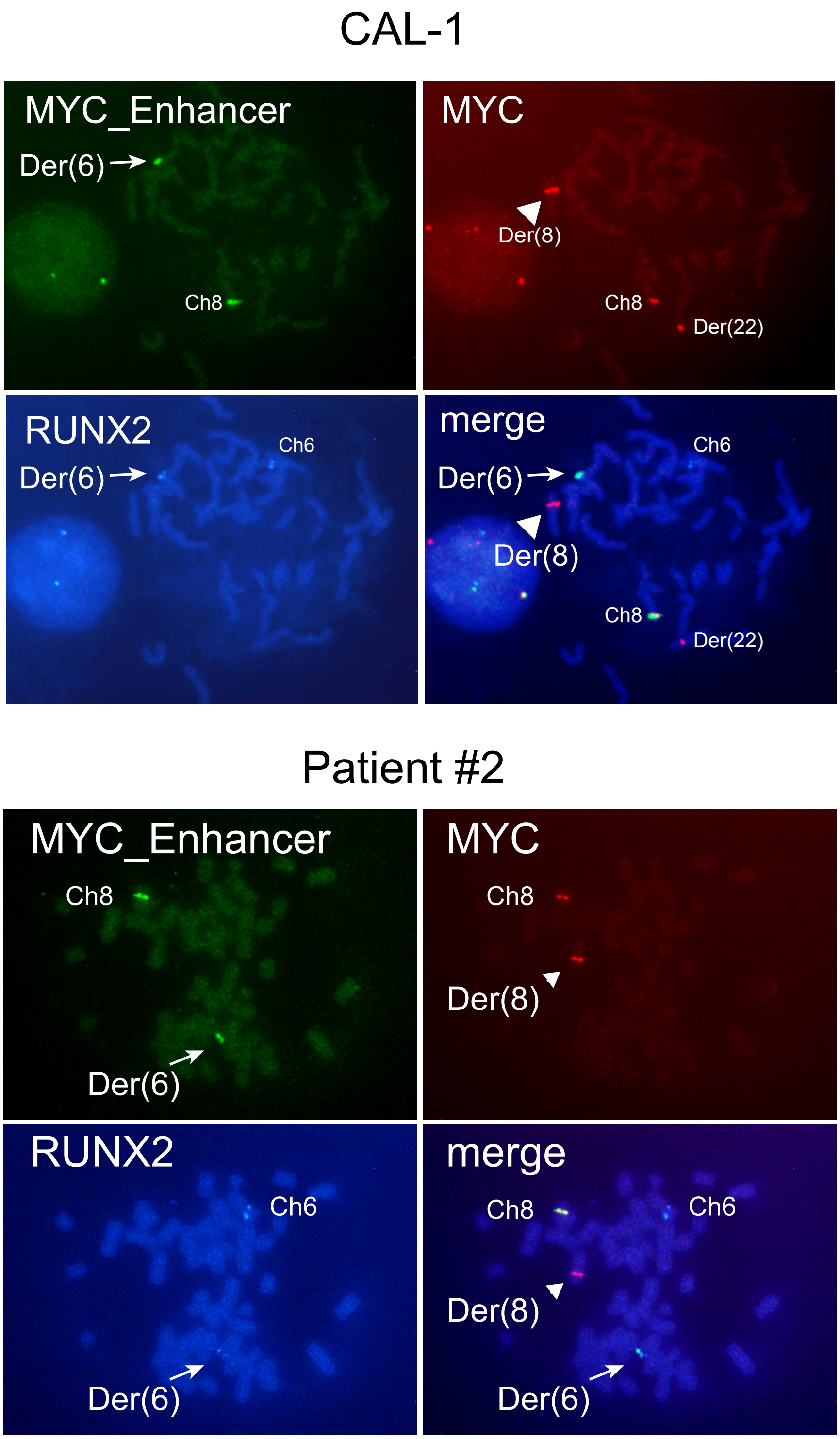

Supplementary Figure 2. Metaphase FISH analysis of BPDCN cells. We found a merged signal of RUNX2 and the enhancer region of MYC on Der(6) and single signals of the MYC on Der(8) and Der(22) in CAL-1 and that on Der(8) in a patient (#2).

Supplementary Figure 3

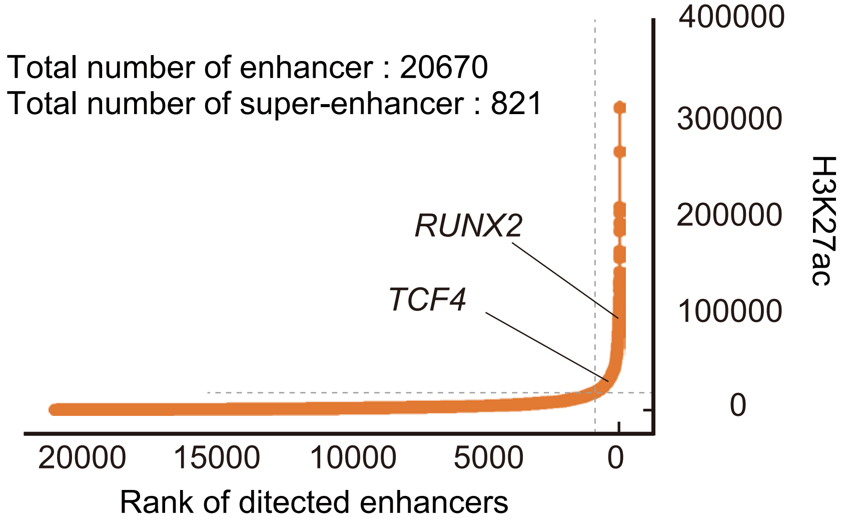

Supplementary Figure 3. Super enhancers of RUNX2 and TCF4 identified by a ROSE analysis for H3K27ac-ChIP sequencing in CAL-1 cells

Supplementary Figure 4

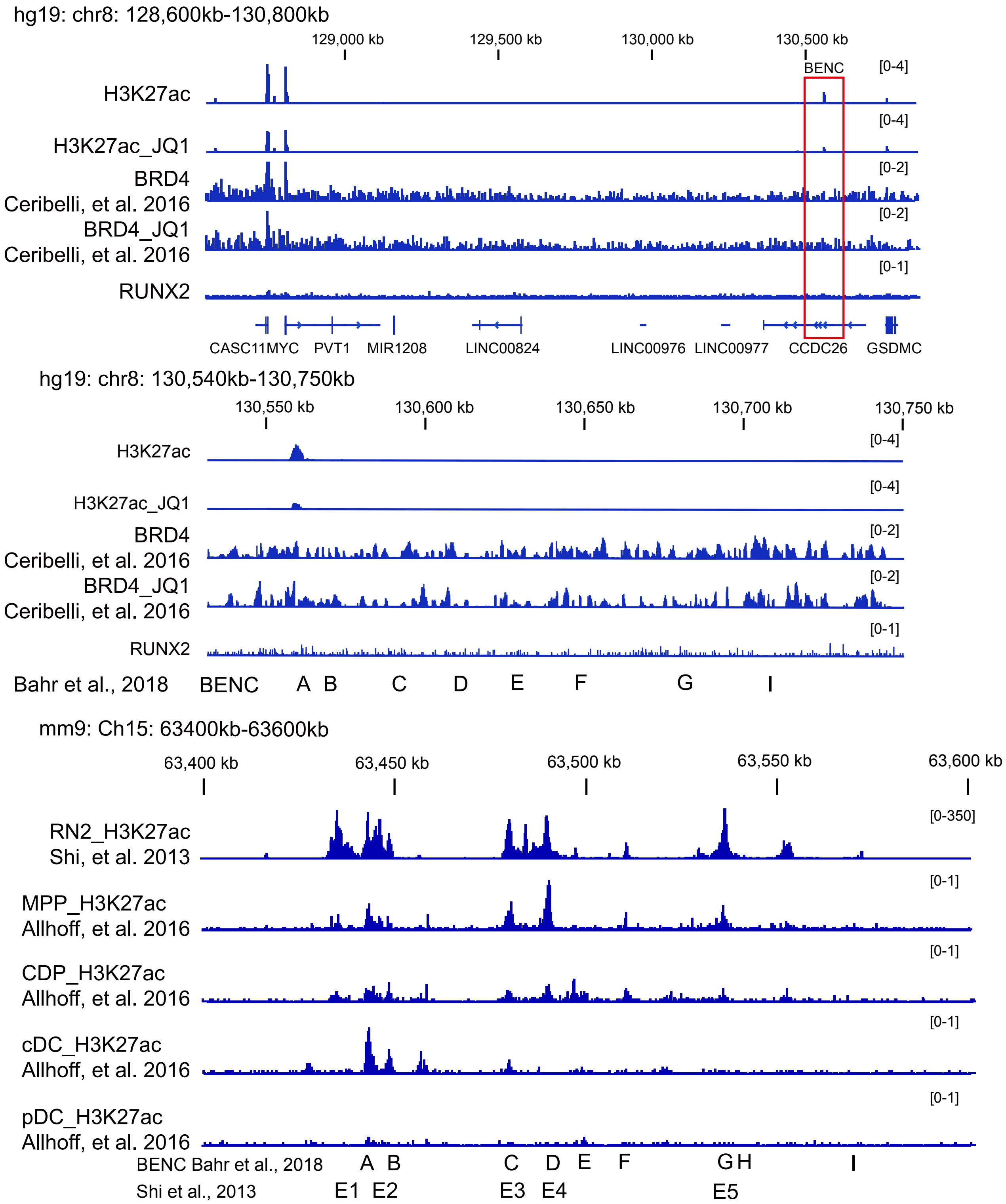

Supplementary Figure 4. Human BPDCN CAL-1 cells showing a small and isolated enhancer of MYC (hg19: chr8; 130558kb-130561kb) defined by ChIP sequencing utilizing either an anti-H3K27ac in this study or anti-BRD4 antibody with or without JQ1 treatment[12] and low enrichments of RUNX2 in the BENC (upper and middle panels), and murine cells showing the BENC enhancer of MYC (mm9: chr15; 63400kb-63600kb) defined by H3K27ac ChIP-sequencing performed on MPPs, CDPs, cDCs, pDCs, and RN2 AML cells (lower panel)[30 31]

Supplementary Figure 5

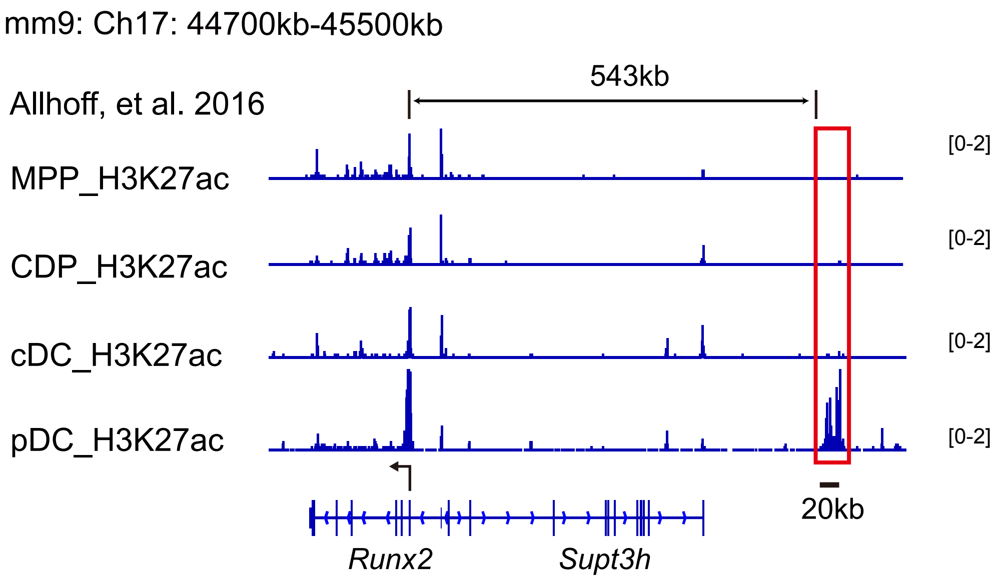

Supplementary Figure 5. Murine pDCs showing a long and clustered enhancer of Runx2 defined by H3K27ac-ChIP sequencing data performed on murine MPP, CDP, cDC, and pDC cells [31]

Supplementary Figure 6

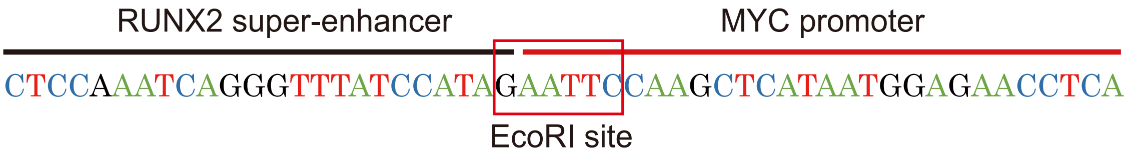

Supplementary Figure 6. Identification of association between the RUNX2 super-enhancer and the MYC promoter in CAL-1 cells

Supplementary Figure 7

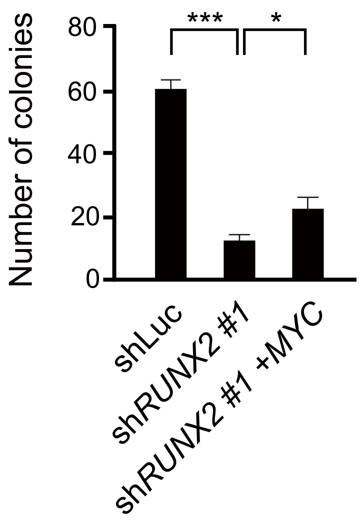

Supplementary Figure 7. Ectopic expression of MYC rescuing the reduced colony formation capacity in RUNX2 KD CAL-1 cells

Supplementary Figure 8

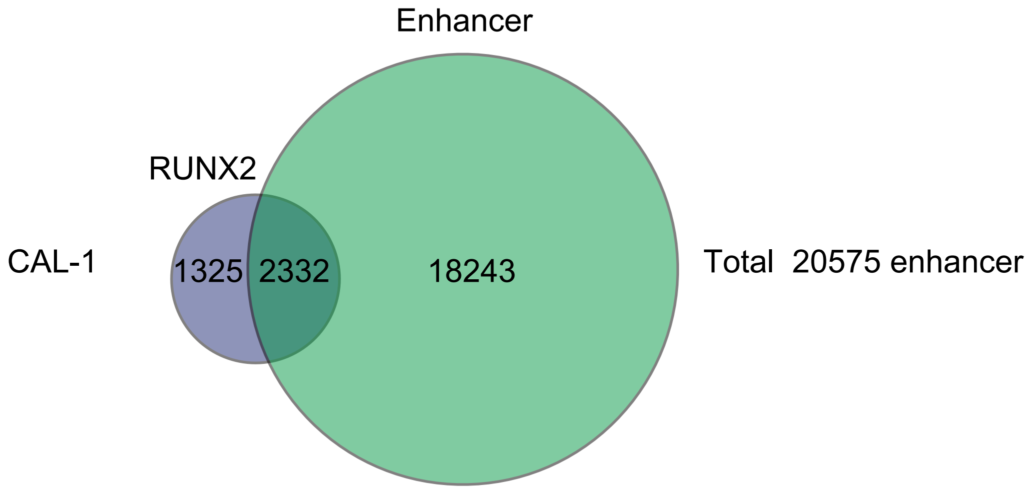

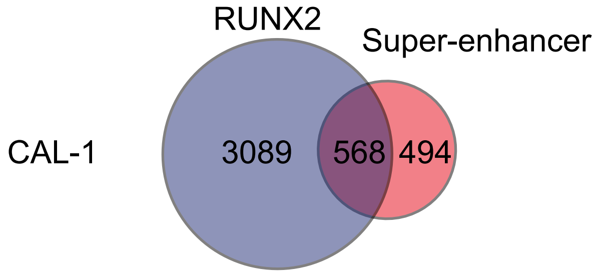

Supplementary Figure 8. Venn diagrams showing the overlaps between RUNX2 binding regions and enhancers or super-enhancers in CAL-1 cells

Supplementary Figure 9

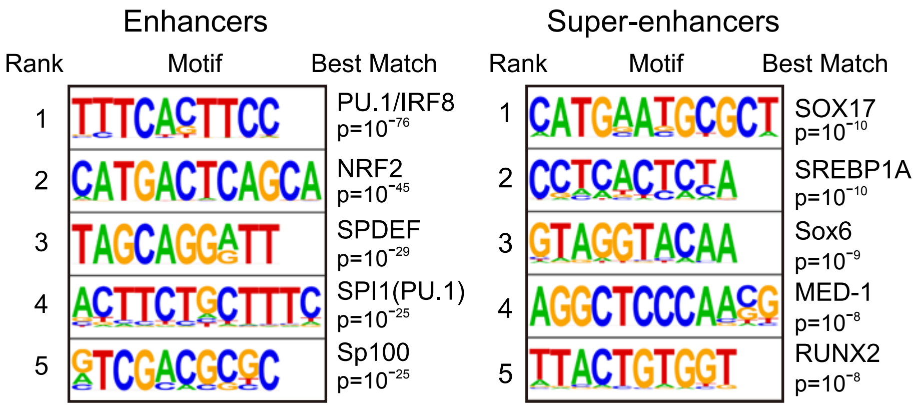

Supplementary Figure 9. Significant enrichment of the RUNX2-binding sequence motif within super-enhancers rather than enhancers in CAL-1 cells

Supplementary Figure 10

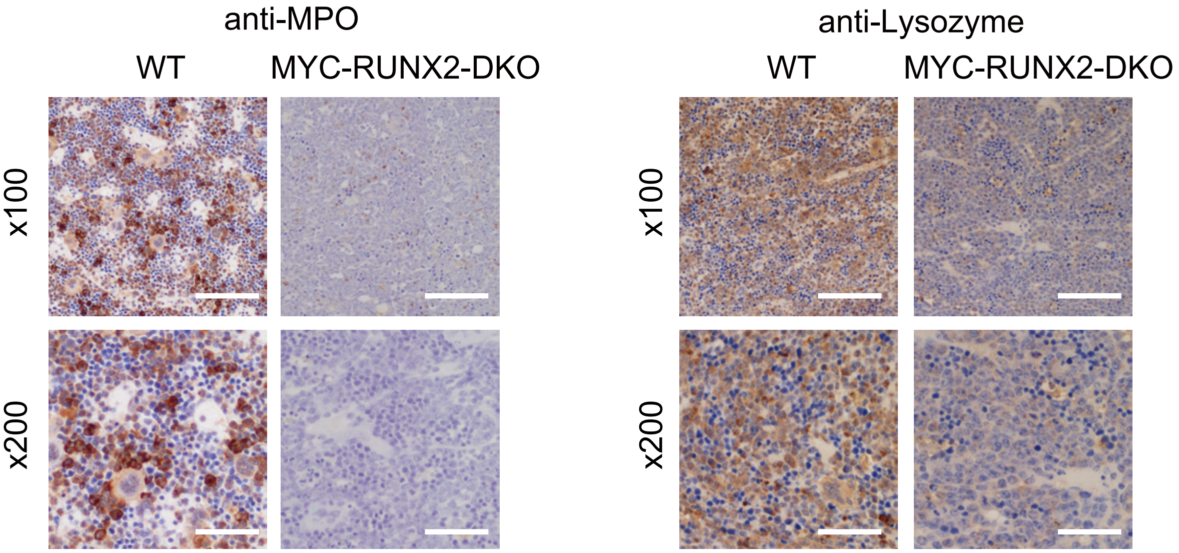

Supplementary Figure 10. Representative histology of the bone marrow tissues of WT and MYC+RUNX2-DKO BPDCN mouse stained with an anti-myeloperoxidase antibody or an anti-lysozyme antibody. Scar bars, 50μm or 100μm.

Supplementary Figure 11

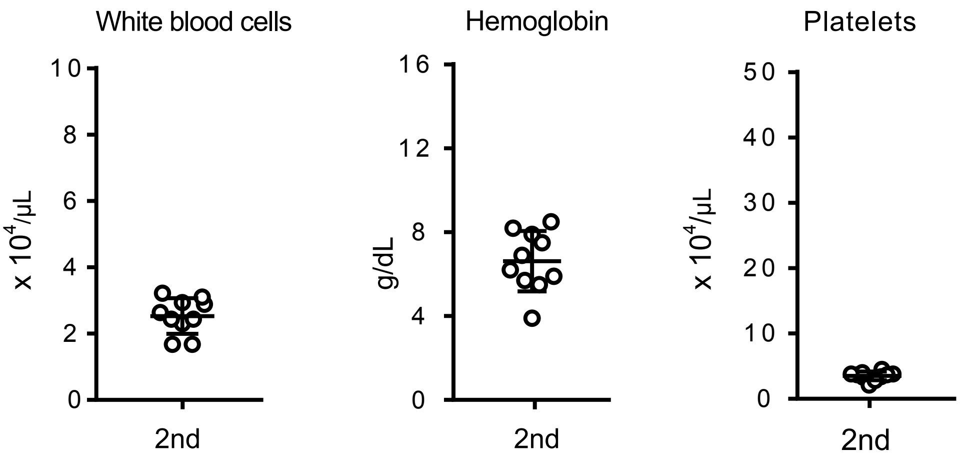

Supplementary Figure 11. Complete blood counts of the secondary-transplanted mice with MYC+RUNX2-DKO cells (n=10). Bars show the mean ± SD and data were combined from 2 independent experiments.

Supplementary Figure 12

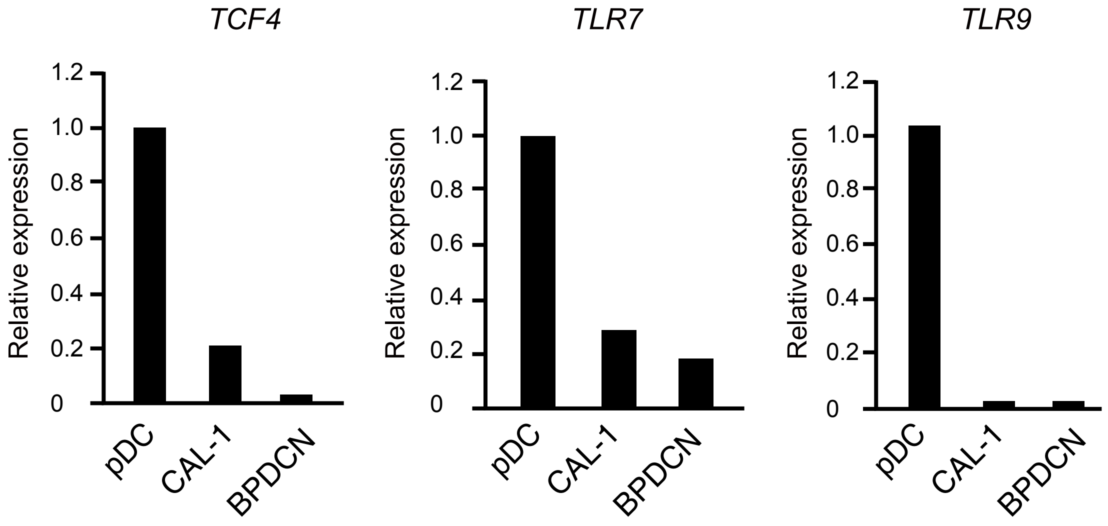

Supplementary Figure 12. Primary BPDCN cells and CAL-1 cells showing lower expression levels of pDC-signature genes such as TCF4, TLR7, and TLR9 than normal pDCs in humans (GSE62014)

Supplementary Figure 13

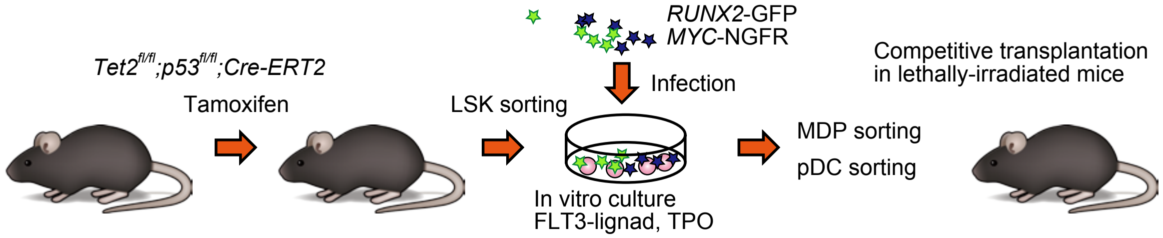

Supplementary Figure 13. Experimental scheme of transplantation of purified MDPs and pDCs from MYC+RUNX2-DKO cells

Supplementary Figure 14

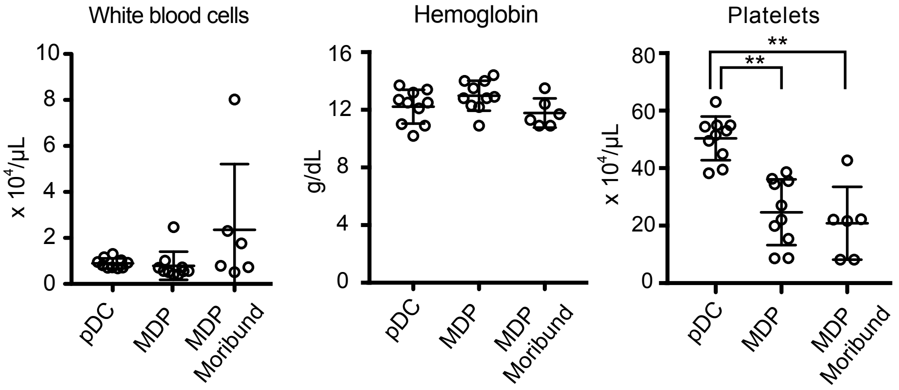

Supplementary Figure 14. Complete blood cell counts of MYC+RUNX2-DKO pDCs (n=10) and MYC+RUNX2-DKO MDPs (n=10) mice 3 weeks after transplantation and moribund MDP leukemic mice (n=6) at the time of sacrifice. Bars show the mean ± SD.

Supplementary Figure 15

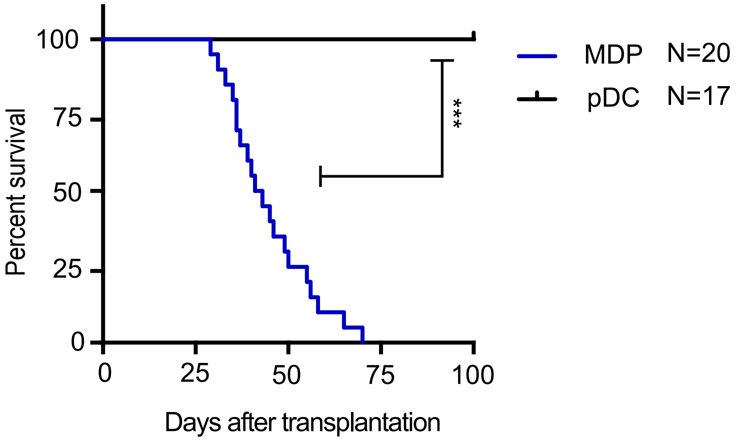

Supplementary Figure 15. Significantly shorter median survival of MYC+RUNX2-DKO MDPs mice (n=20) (42 days versus undetermined for MYC+RUNX2-DKO pDCs mice (n=17); ***p<0.0001 by the Log-rank test). Data were combined from 2 independent experiments.

Supplementary Figure 16

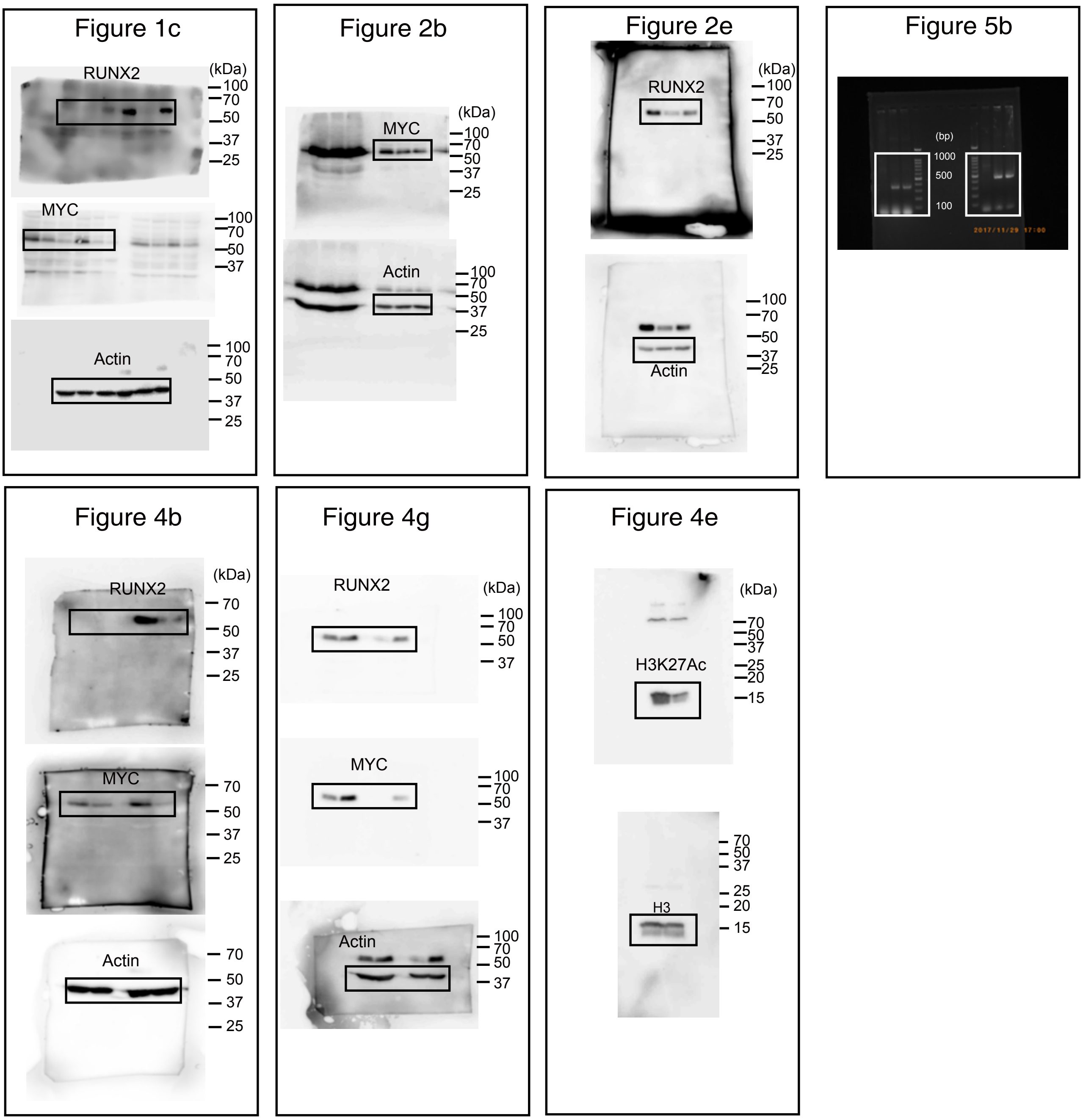

Supplementary Figure 16. Original images of gels and blots

Supplementary Table 1

Gene set \ Sample	BPDCN versus LMPPs	BPDCN versus GMPs	BPDCN versus MDPs	BPDCN versus pDCs
MYC_target_v1	NES=3.176 FDR=0.000	NES=2.815 FDR=0.000	NES=2.490 FDR=0.000	NES=2.490 FDR=0.000
pDC-signature-gene	NES=1.677 FDR=0.000	NES=1.175 FDR=0.000	NES=2.353 FDR=0.000	NES=-2.928 FDR=0.000
Inflamatory_response	NES=-1.607 FDR=0.000	NES=-1.841 FDR=0.000	NES=-1.749 FDR=0.000	NES=-2.065 FDR=0.000
Interferonα_response	NES=-1.759 FDR=0.000	NES=-1.558 FDR=0.006	NES=-1.528 FDR=0.000	NES=-2.088 FDR=0.000

Supplementary Table 1. GSEA plots for Hallmark MYC targets V1, pDC-signature genes, Hallmark inflammatory response and Hallmarks interferon α response comparing MYC+RUNX2-DKO leukemic cells to LMPPs, MDPs, GMPs, and pDCs isolated from wild-type mice. Normalized enrichment score (NES) and false discovery rate (FDR) q-values are indicated.

Supplementary Table 2

q-PCR	5'- sequence -3'
RUNX2-F	TCCCTGAACTCTGCACCAAG
RUNX2-R	ATCTGGCTCAGGTAGGAGGG
MYC-F	AATGAAAAGGCCCCCAAGGTAGTTATCC
MYC-R	GTCGTTTCCGCAACAAGTCCTCTTC
TCF4-F	ACCAACAGCGAATGGCTGCCTTA
TCF4-R	TCCCACTGCTCACAGGAGGTGAA
TLR9-F	GGGACCTCGAGTGTGAAGCATCC
TLR9-R	CATGATGGCCTGCACCAGGAGAG
TLR7-F	TGCTCTGCTCTCTTCAACCAGACC
TLR7-R	ACCATCTAGCCCCAAGGAGTTTGG
IL3-RA-F	CCCCATCGGTGACAGCTTCCAAA
IL3-RA-R	CACAAGCCCTGAACCCCAGTCTC

3C-qPCR	5'- sequence -3'
RUNX2-promoter	CTGTCACACTGAGCTTGACACCGC
RUNX2-super-enhancer	GCATATTTAACACAGTGCAACAGCC
MYC-promoter	CCATGGTCCAAAATGAGGTTCTCC
RUNX2-super-enhancer-seq	CTTGCTACGACCTTTGGTGTCC
MYC-promoter-seq	GGTTGAGAATCCCTGGGTTCACTCC

sgRNA	5'- sequence -3'
sgRNA1	TTCTGCGTCGTTCACGCTGGGGG
sgRNA2	TAGCTGCTGGCGATCCAGCAGGG
sgRNA3	TCAGTAGACGTTGAACTGGACGG

Genomic PCR	5'- sequence -3'
check-deletion-gRNA#1	GTGTGCTGTTTGCTAAGACTGCTGG
check-deletion-gRNA#2	CTTAGCACAGAGGAATAAGCCC
check-deletion-gRNA#3	CCTTCTAGGGTCCATGAGTAACCC

Supplementary Table 2. List of primers for genomic PCR and quantitative RT-PCR
