# Supplementary material for: Lineage-specific RUNX2 super-enhancer activates MYC and promotes the development of blastic plasmacytoid dendritic cell neoplasm
Source: Nat Commun. 2019 Apr 10;10:1653. doi: 10.1038/s41467-019-09710-z (PMC6458132; doi:10.1038/s41467-019-09710-z)
Supplement: Supplementary file 3 — Description of Additional Supplementary Files [file 41467_2019_9710_MOESM3_ESM.pdf]

Description of Additional Supplementary Files

File Name: Supplementary Data 1
Description: List of up-regulated genes and down-regulated genes in RUNX2 KD-CAL-1 cells

File Name: Supplementary Data 2
Description: List of up-regulated genes and down-regulated genes in BPDCN cells versus normal pDCs in humans and mice
