# Supplementary material for: Lineage-specific RUNX2 super-enhancer activates MYC and promotes the development of blastic plasmacytoid dendritic cell neoplasm
Source: Nat Commun. 2019 Apr 10;10:1653. doi: 10.1038/s41467-019-09710-z (PMC6458132; doi:10.1038/s41467-019-09710-z)
Supplement: Supplementary file 6 — Reporting Summary [file 41467_2019_9710_MOESM6_ESM.pdf]

nature research

Corresponding author(s): Goro Sashida

Last updated by author(s): Mar 12, 2019

Reporting Summary

Nature Research wishes to improve the reproducibility of the work that we publish. This form provides structure for consistency and transparency in reporting. For further information on Nature Research policies, see Authors & Referees and the Editorial Policy Checklist.

Statistics

For all statistical analyses, confirm that the following items are present in the figure legend, table legend, main text, or Methods section.

n/a	Confirmed	
☐	☒	The exact sample size (*n*) for each experimental group/condition, given as a discrete number and unit of measurement
☐	☒	A statement on whether measurements were taken from distinct samples or whether the same sample was measured repeatedly
☐	☒	The statistical test(s) used AND whether they are one- or two-sided<br>*Only common tests should be described solely by name; describe more complex techniques in the Methods section.*
☐	☒	A description of all covariates tested
☐	☒	A description of any assumptions or corrections, such as tests of normality and adjustment for multiple comparisons
☐	☒	A full description of the statistical parameters including central tendency (e.g. means) or other basic estimates (e.g. regression coefficient) AND variation (e.g. standard deviation) or associated estimates of uncertainty (e.g. confidence intervals)
☐	☒	For null hypothesis testing, the test statistic (e.g. *F*, *t*, *r*) with confidence intervals, effect sizes, degrees of freedom and *P* value noted<br>*Give P values as exact values whenever suitable.*
☐	☒	For Bayesian analysis, information on the choice of priors and Markov chain Monte Carlo settings
☐	☒	For hierarchical and complex designs, identification of the appropriate level for tests and full reporting of outcomes
☒	☐	Estimates of effect sizes (e.g. Cohen's *d*, Pearson's *r*), indicating how they were calculated

Our web collection on statistics for biologists contains articles on many of the points above.

Software and code

Policy information about availability of computer code

Data collection	Bowtie2, version 2.2.6
Data analysis	HOMER, version 4.9; BWA-MEM; Graph Pad Prism, version 7; Gene Set Enrichment Analysis; Heatmap2; CLC workbench

For manuscripts utilizing custom algorithms or software that are central to the research but not yet described in published literature, software must be made available to editors/reviewers. We strongly encourage code deposition in a community repository (e.g. GitHub). See the Nature Research guidelines for submitting code & software for further information.

Data

Policy information about availability of data

All manuscripts must include a data availability statement. This statement should provide the following information, where applicable:
- Accession codes, unique identifiers, or web links for publicly available datasets
- A list of figures that have associated raw data
- A description of any restrictions on data availability

Whole genome sequencing, DRA006440; Microarray, GSE110140; ChIP-sequencing, DRA006440 and DRA007469; RNA-sequencing, DRA006565

Field-specific reporting

Please select the one below that is the best fit for your research. If you are not sure, read the appropriate sections before making your selection.

☒ Life sciences ☐ Behavioural & social sciences ☐ Ecological, evolutionary & environmental sciences

For a reference copy of the document with all sections, see nature.com/documents/nr-reporting-summary-flat.pdf

Life sciences study design

All studies must disclose on these points even when the disclosure is negative.

Sample size	No statistical methods were used to predetermine sample size for animal studies.
Data exclusions	Mice were excluded from the analysis if blood cells were not recovered 2 weeks post transplantation due to injection failure.
Replication	All Data were confirmed by 2 to 4 independent experiments.
Randomization	All mice experiments were performed without randomization.
Blinding	All mice experiments were performed without blinding.

Reporting for specific materials, systems and methods

We require information from authors about some types of materials, experimental systems and methods used in many studies. Here, indicate whether each material, system or method listed is relevant to your study. If you are not sure if a list item applies to your research, read the appropriate section before selecting a response.

Materials & experimental systems

n/a	Involved in the study	
☐	☒	Antibodies
☐	☒	Eukaryotic cell lines
☒	☐	Palaeontology
☐	☒	Animals and other organisms
☐	☒	Human research participants
☒	☐	Clinical data

Methods

n/a	Involved in the study	
☐	☒	ChIP-seq
☐	☒	Flow cytometry
☒	☐	MRI-based neuroimaging

Antibodies

Antibodies used	anti-Annexin V–APC antibody (BD 550474), anti-BrdU-APC antibody (BD), anti-human antibodies (clone and catalogue numbers): CD271 (ME20.4, 345108), CD45 (HI30, 25-0459), CD4 (OKT4, 50-0048), CD56 (MY31, 60-0564), CD123 (6H6, 306011), and TCL1 (1-21, 330506), as well as the following anti-murine antibodies (clone and catalogue numbers): CD45.2 (104, 109820), CD45.1 (A20, 110730), Gr1 (RB6-8C5, 108404), CD11b/Mac1 (M1/70, 101208), CD11c (N418, 117304), NK1.1 (PK136, 108704), Ter119 (116204), CD127/IL-7Rα (A7R34, 121104), Bst2 (927, 127010), B220 (RA3-6B2, 103212), CD3e (145-2C11, 100320), CD4 (L3T4, 100526), CD8α (53-6.7, 100714), CD56 (809220, FAB7820A), CD117/c-Kit (2B8, 105812), Sca1 (D7, 108114), CD34 (MEC14.7, 11-0341-85), CD123 (5B11, 106005), CD135 (A2F10, 135306), CD115 (AFS98, 135510), and FcγRII-III (93, 101308).
Validation	Validation statements of manufacturers' websites (BD, BioLegend, eBioscience, R&D systems or TONBO biosciences)

Eukaryotic cell lines

Policy information about cell lines

Cell line source(s)	CAL-1, Jurkat, HEL, MOLM-13, MONO-MAC-1, U2OS, and Saos2
Authentication	CAL-1 is not authenticated.
Mycoplasma contamination	CAL-1 is not tested.
Commonly misidentified lines (See ICLAC register)	not listed

Animals and other organisms

Policy information about studies involving animals; ARRIVE guidelines recommended for reporting animal research

Laboratory animals	All mice were in the C57BL/6 background. p53flox/flox, Tet2flox/flox, and Rosa26:Cre-ERT2 were used in this study.
Wild animals	This study did not use wild animals.
Field-collected samples	This study did not use those samples.

Ethics oversight | All mice experiments were approved by the Review Board for Animal Experiments of Kumamoto University (Kumamoto, Japan).

Note that full information on the approval of the study protocol must also be provided in the manuscript.

Human research participants

Policy information about studies involving human research participants

Population characteristics | Clinical diagnosis of BPDCN of patients

Recruitment | Biospecimens (blood cells) were obtained after written consent was obtained from patients.

Ethics oversight | The Institutional Review Committees at Kumamoto University (Kumamoto, Japan) and Tokyo Medical University (Tokyo, Japan).

Note that full information on the approval of the study protocol must also be provided in the manuscript.

ChIP-seq

Data deposition

☒ Confirm that both raw and final processed data have been deposited in a public database such as GEO.

☐ Confirm that you have deposited or provided access to graph files (e.g. BED files) for the called peaks.

Data access links
May remain private before publication. | RUNX2-ChIP-seq : https://ddbj.nig.ac.jp/DRASearch/submission?acc=DRA007469
H3K27ac-ChIP-seq :https://ddbj.nig.ac.jp/DRASearch/submission?acc=DRA006440

Files in database submission | RUNX2-ChIP: DRR154170, DRR154171 H3K27ac-ChIP-seq : DRR120855, DRR120856, DRR120857, DRR120858

Genome browser session (e.g. UCSC) | *Provide a link to an anonymized genome browser session for "Initial submission" and "Revised version" documents only, to enable peer review. Write "no longer applicable" for "Final submission" documents.*

Methodology

Replicates | Pooled specimens in each sample

Sequencing depth | Type: Single end; Total reads: about 30-50 million per sample; Mapped reads: about 60-70% of total reads per sample

Antibodies | anti-H3K27ac antibody (Active motif, MABI 0309) and anti-RUNX2 antibody (MBL, 8G5)

Peak calling parameters | Default setting of HOMER software: FDR rate threshold = 0.001000, Fold over local region required = 4.00

Data quality | RUNX2 ChIP-seq detected about 3600 peaks, and H3K27ac ChIP-seq detected about 20600 peaks by HOMER default setting.

Software | Bowtie2, HOMER, bedtools, ROSE, MACS2, IGV

Flow Cytometry

Plots

Confirm that:

☒ The axis labels state the marker and fluorochrome used (e.g. CD4-FITC).

☒ The axis scales are clearly visible. Include numbers along axes only for bottom left plot of group (a 'group' is an analysis of identical markers).

☒ All plots are contour plots with outliers or pseudocolor plots.

☒ A numerical value for number of cells or percentage (with statistics) is provided.

Methodology

Sample preparation | All samples were freshly isolated from sacrificed mice and immediately stained by antibodies.

Instrument | FACSAriaII, FACSCantoII (BD)

Software | FlowJo version 10 (FlowJo); FACSDIVA (BD)

Cell population abundance | Sorted samples were examined by FACS analysis or cytologic evaluation following MG staining.

Gating strategy	After excluding doublets on FFS/SSC, propidium iodide-negative alive cells were applied for gating.

☐ Tick this box to confirm that a figure exemplifying the gating strategy is provided in the Supplementary Information.
